# Orthodontic camouflage as a treatment alternative for skeletal Class III

**DOI:** 10.1590/2177-6709.26.4.e21bbo4

**Published:** 2021-09-10

**Authors:** Monica Tirre de Souza ARAUJO, Luciana Rougemont SQUEFF

**Affiliations:** 1 Universidade Federal do Rio de Janeiro (Rio de Janeiro/RJ, Brazil).

**Keywords:** Class III malocclusion, Orthodontic camouflage, Dental compensation

## Abstract

**Introduction:**

Skeletal Class III malocclusion is a deformity of complex treatment, with few intervention alternatives, which are further limited in nongrowing patients. In most cases, orthognathic surgery is the ideal treatment for adults, an option often refused by patients. Mild to moderate skeletal Class III malocclusions and acceptable facial esthetics can benefit from a course of treatment in which dental movements are used to compensate for the skeletal discrepancy.

**Objective:**

This study aimed to discuss orthodontic camouflage as an option for adult patients with Class III malocclusion, emphasizing its indications, implications and expected results.

## RESUMO

**Introdução:**

A má oclusão esquelética de Classe III é uma deformidade de difícil tratamento e com poucas alternativas de intervenção, que ficam ainda mais limitadas em pacientes sem crescimento. Na maior parte dos casos, o tratamento ideal para adultos é a cirurgia ortognática, opção muitas vezes recusada pelo paciente. As más oclusões esqueléticas de Classe III leve a moderada e com estética facial aceitável podem se beneficiar de um plano de tratamento no qual movimentações dentárias são realizadas para compensar a discrepância esquelética.

**Objetivo:**

O objetivo do presente estudo foi discutir a camuflagem ortodôntica como opção para pacientes adultos com má oclusão de Classe III, ressaltando suas indicações, implicações e resultados esperados.

## INTRODUCTION

Skeletal Class III malocclusion is one of the most challenging problems faced by orthodontists.^1^ ^,^ ^2^ It is characterized by an anteroposterior discrepancy between the maxilla and the mandible usually associated with dentoalveolar compensation (protruded maxillary incisors and/or proclined and retroclined mandibular incisors), to maintain the function and camouflage the existing skeletal discrepancy.^3^ Compromised facial esthetics is often present, constituting, in these cases, the main reason why patients or their guardians seek treatment.^4^

In adult patients, treatment is more complex due to the limited options available.^5^ In most cases, orthodontic treatment combined with orthognathic surgery is often the ideal treatment. However, many patients refuse the surgical option due to its cost or the invasive nature of the procedure.^6^ ^,^ ^7^

Nongrowing patients with mild to moderate skeletal Class III malocclusion and acceptable facial esthetics can benefit from camouflage orthodontic treatment,^3^ to enable the displacing of teeth relative to their supporting bone to compensate for an underlying jaw discrepancy. It is indicated when growth modification to overcome the basic problem is not feasible.^1^ The objectives of camouflage treatment include attaining acceptable occlusion, function, and esthetics through dentoalveolar compensation for the skeletal discrepancy.^8^

Camouflage treatment was introduced into orthodontics in the 1930s and 1940s, when extraction to camouflage a skeletal malocclusion became popular, as growth modification had been widely regarded as ineffective, and surgical correction was still in early development. The strategy to camouflage a Class III malocclusion usually involves proclination of the maxillary incisors and retroclination of the mandibular incisors, to improve dental occlusion, although it might not correct the skeletal problem or facial profile.^1^

In patients with moderate skeletal Class III malocclusion, the decision for orthodontic camouflage as a treatment option should consider some parameters. First, the extent of compromise of facial esthetics must be assessed and how important this is for the patient. In cases of significant esthetic complaint, orthognathic surgery is required.^9^ ^,^ ^10^ The second parameter is the anteroposterior position and inclination of maxillary and mandibular incisors, and whether their orthodontic movement is sufficient for correcting the malocclusion. The third parameter is the thickness of mandibular symphysis, which should allow extensive incisor retraction. Finally, the degree of anteroposterior discrepancy must also be assessed. Even if facial esthetics is acceptable, the symphysis is thick enough, and the mandibular incisors are favorably inclined, camouflage will not be indicated if the anteroposterior discrepancy is too severe.^10^ ^,^ ^11^

The initial positioning of maxillary and mandibular anterior teeth, and mandibular growth are unfavorable for the nonsurgical treatment of Class III malocclusion. Maxillary incisors showing compensatory protrusion and mandibular incisors showing lingual inclinations are often observed, limiting the amount of negative overjet that could be treated without surgery.^13^

From this perspective, this study aimed to address orthodontic camouflage as an option for adult patients with skeletal Class III malocclusion, and describe the orthodontic treatment of a male patient with 19 years and 8 months, treated with dental compensations - this case was submitted to the Brazilian Board of Orthodontics (BBO).

## CASE REPORT

A male patient (19 years and 8 months) in good general health sought orthodontic treatment with the complaint of being dissatisfied with the result obtained after eight years of treatment, dissatisfied with his smile, and bothered by the lower crowding. He also complained about not having the upper left canine (#23), lost during previous orthodontic treatment (resulting from eruption disturbance). 

Facial examination revealed facial asymmetry and increased lower third of the face. Smile esthetics was damaged by posterior crossbite, which widened his buccal corridor. The facial profile was concave, with a slight deficiency in the middle third of the face and a good nasolabial angle. Intraoral clinical examination revealed that the patient, although without a history of carious lesions or dental restorations, presented poor oral hygiene. The patient had Angle’s Class III malocclusion, more pronounced on the right side, with reduced overbite and overjet, and open bite in the region of teeth #12, #13, #22 and #24. Bilateral posterior crossbite was more extensive on the left side, and tooth #23 was missing, creating spaces and generating significant asymmetry in the maxillary arch. Moreover, according to his complaint, the patient showed a 10 mm mandibular crowding in the anterior and middle region, with retroclined mandibular incisors, compensating the Class III malocclusion, and marked gingival recession in tooth #33. Finally, there was a lack of space in the posterior region for the correct positioning of teeth #36 and #46. 

**Figure 1:**
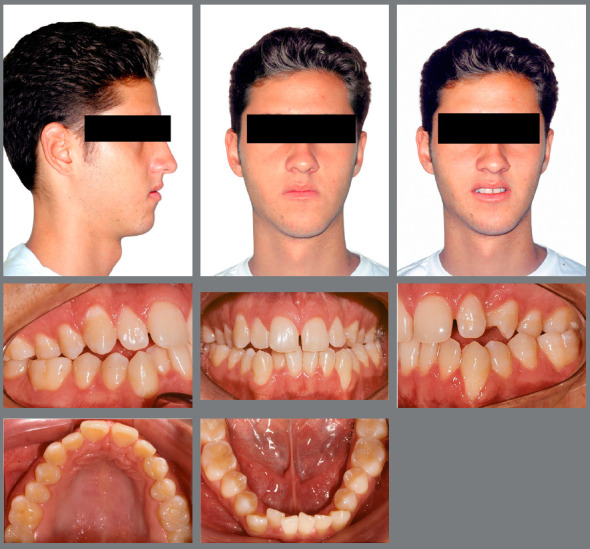
Initial facial and intraoral photographs.

**Figure 2:**
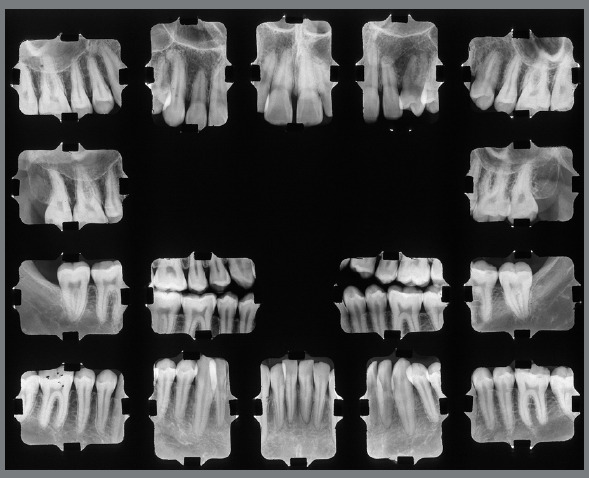
Initial periapical radiographs.

**Figure 3:**
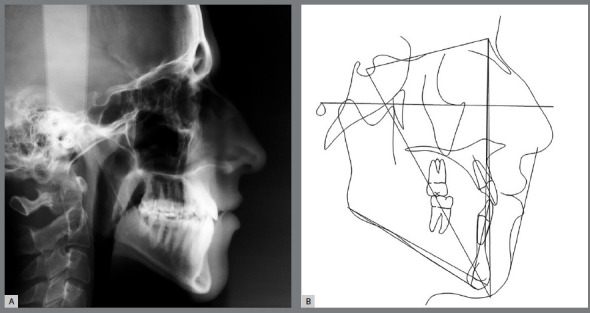
Initial cephalometric profile radiograph (A) and cephalometric tracing (B).

Functional examination revealed the absence of lateral disocclusion, compromised by the missing #23, occasional TMJ clicking, slightly atypical swallowing and phonation, and mouth breathing (Figs 1 and 2). Initial lateral cephalometric radiograph revealed a sagittal maxillary deficiency (SNA = 76) and a clear Class III skeletal pattern, with ANB = 0. Moreover, vertical growth deficiency of the mandibular ramus and a vertical growth pattern (SN.GoGn = 43 ^o^ and FMA = 31^o^) were observed, with compensatory inclination of the incisors (1.NA = 25^o^, 1.NB = 12^o^and IMPA = 70^o^) and a concave profile (NAPog = -2°) (Fig 3, Table 1).

**Table 1:** Initial (A), final (B) and 8 years after treatment (C) cephalometric values.

	MEASURES		Normal	A	B	A/B	C
Skeletal pattern	SNA	(Steiner)	82°	75.4°	75.9°	0.5°	78.9°
	SNB	(Steiner)	80°	75.1°	75.5°	0.4°	78.2°
	ANB	(Steiner)	2°	0.2°	0.4°	0.2°	0.7°
	Wits	(Jacobson)	♀ 0 ±2mm ♂ 1 ±2mm	-5.4 mm	-2.7 mm	2.7 mm	-6.0 mm
	Angle of convexity	(Downs)	0°	-2.3°	- 3.0°	1.3°	-1.0°
	Y-Axis	(Downs)	59°	60.4°	57.4°	3.0°	64.6°
	Facial Angle	(Downs)	87°	91.4°	94.5°	3.1°	89.2°
	SN.GoGn	(Steiner)	32°	43°	43.3°	0.3°	42.3°
	FMA	(Tweed)	25°	29°	28°	1°	34°
Dental pattern	IMPA	(Tweed)	90°	73°	61°	12°	60°
	1.NA (degrees)	(Steiner)	22°	25.2°	23.9°	1.3°	19.5°
	1-NA (mm)	(Steiner)	4 mm	5.4 mm	3.4 mm	2 mm	1.5 mm
	1.NB (degrees)	(Steiner)	25°	11.6°	5.8°	5.8°	3.8°
	1-NB (mm)	(Steiner)	4mm	3.2mm	1.1mm	1.4°	0 mm
	- Interincisal angle	(Downs)	130°	142.9°	149.9°	7°	156°
	1 - APg	(Ricketts)	1mm	1.3 mm	-0.7 mm	2.0 mm	-1.5 mm
Profile	Upper Lip - Line S	(Steiner)	0mm	-2.2 mm	-4.0 mm	1.8 mm	-3.2 mm
	Lower Lip - Line S	(Steiner)	0mm	0.5 mm	-5.1 mm	4.6 mm	-3.9 mm

## TREATMENT PLAN AND MECHANICS USED

Since he was satisfied with his facial esthetics, the patient was contrary to any surgical intervention to treat the skeletal Class III malocclusion. Therefore, the chosen treatment was orthodontic camouflage, with the extraction of tooth #15 followed by the loss of upper anchorage and extraction of the first mandibular premolars, maintaining maximum anchorage for the retraction of the anterior teeth without modifying the positions of teeth #36 and #46.

The objectives of treatment included the improvement of oral hygiene, with health instructions and rigorous dental plaque control, to prevent the worsening of gingival recession in tooth #33. Profile harmonization in the lower third and negative discrepancy in the mandibular arch were favored by the extraction of the first premolars and retraction of the mandibular incisors, whereas the positive discrepancy and asymmetry in the maxillary arch was corrected by closing the spaces and with the extraction of tooth #15. The correction of the transverse problem caused by bilateral posterior crossbite by improving the shape of the maxillary arch was well planned, as well as the vertical control for improving or, at least, maintaining the existing counterclockwise rotation. The patient was referred to speech therapy and otorhinolaryngology services to correct slightly atypical swallowing and phonation habits and mouth breathing. 

Treatment began by mounting a fixed standard edgewise appliance with 0.022 x 0.028-in slots. A lingual arch was placed in the mandibular arch as an anchorage resource, supported by the mandibular first molars. Next, the alignment and leveling phase was started. Stainless steel archwires from 0.014-in to 0.020-in were used in the mandibular arch, followed by 0.019 x 0.026-in archwire, loss of upper posterior anchorage with elastomeric chain, realignment, and a final 0.019 x 0.026-in archwire. In the mandibular arch, a 0.014-in archwire was used with a vertical loop mesial to the canines and a teardrop loop in the extraction space, using active tie-back, taking care not to procline the incisors. Treatment followed with a sequence of 0.016-in and 0.018-in contracted stainless steel archwires, starting the distalization of teeth #33 and #43 still in the 0.018-in archwire (passive) with elastomeric chain. At the end of canine retraction, incisor retraction began with the 0.019 x 0.026-in stainless steel coil spring retraction archwire. Finally, continuous 0.019 x 0.026-in archwire was used. As an auxiliary resource, Class III mechanics was used on the right side with intermaxillary elastics, in addition to complementary binary resources to achieve the translation movement. At the end of orthodontic tooth movement, an upper removable wraparound retainer was placed with vertical loops in the region of the canines (kept continuously for one year, and for another five years, just for sleeping), whereas, in the lower arch, a 3x3 fixed lingual retainer was placed (removed seven years later). After removing these appliances, no retention device was installed, and the patient was followed up annually.

## TREATMENT RESULTS

**Figure 4:**
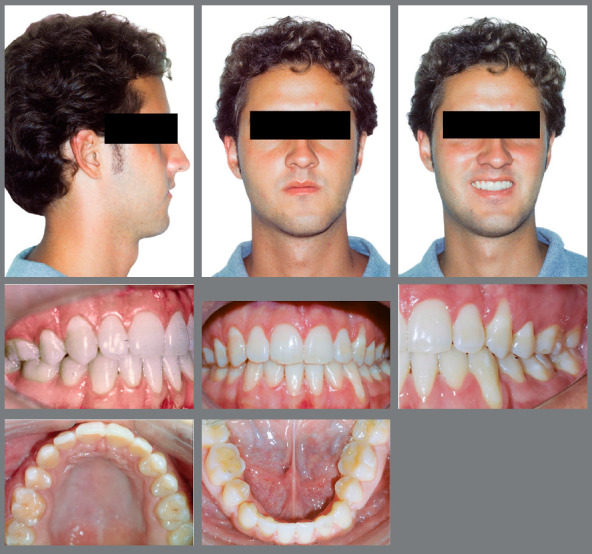
Final facial and intraoral photographs.

**Figure 5:**
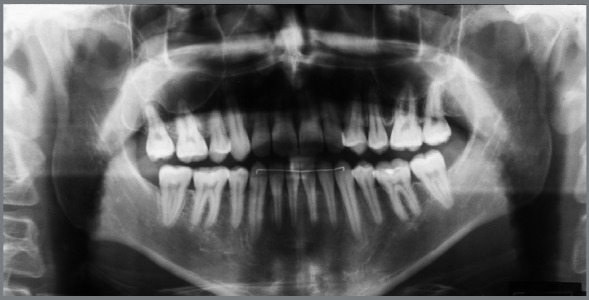
Final panoramic radiograph.

**Figure 6:**
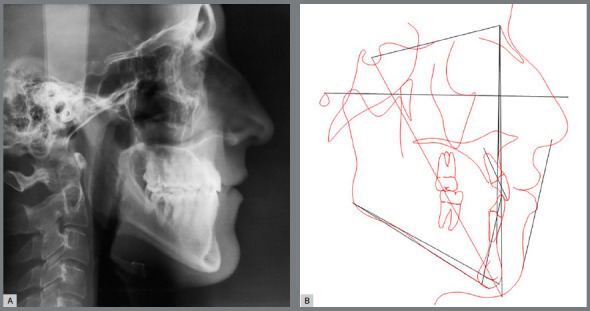
Final cephalometric profile radiograph (A) and cephalometric tracing (B).

The orthodontic treatment performed had its objectives achieved and provided functional and esthetic improvements. The deficiency of the middle third remained, as well as the facial asymmetry, but they remained discreet. The buccal corridor was shortened with the correction of bilateral posterior crossbite, making the smile more pleasant. The positioning of the lower lip was slightly modified, making the patient’s profile more harmonious. The shape of the arches was improved, with the mandibular crowding and spaces in the maxillary arch being eliminated, achieving root parallelism and stability of the gingival recession of tooth #33. On the right side, a Class I relationship of molars and canines was reached. On the left side, a good relationship was obtained between teeth #33 and #24, and a good degree of overbite and overjet was obtained. Good root parallelism was achieved, and the gingival recession in tooth #33 was stabilized. The patient’s skeletal pattern was preserved, and the objective of vertical control was achieved by maintaining the initial anticlockwise rotation (SN.GoGn = 43^o^ and FMA = 29^o^). New dental compensations were required for orthodontic correction (1.NA = 24^o^, 1.NB = 6^o^ and IMPA = 64^o^) and the initially concave profile was maintained (NAPog = -3^o^) (Figs 4 to 6, Table 1).

With regard to the function, tooth guidance was restored, achieving normal functional movements, with posterior disocclusion in protrusion and laterality movements with disocclusion in the balancing side, not requiring the wearing of the palatal cusp of tooth #24. Atypical swallowing, phonation, and mouth breathing were not corrected, as the patient did not seek the therapies recommended throughout orthodontic treatment. 

## DISCUSSION

Patients with severe Class III skeletal deformity are often candidates for orthognathic surgery as the only choice toward normal occlusion and an esthetic profile.^14^However, the dilemma lies in the fact that most patients reject surgical therapy, persisting in orthodontic treatment. Moreover, the facial profile of Class III skeletal deformities is always the primary concern of these patients when seeking treatment. This truly is a great challenge for orthodontists, and estimating facial changes and occlusal improvements is essential when developing a treatment plan.^3^Psychological (instead of morphological) characteristics are probably a factor of significant influence for an individual when deciding about whether or not to accept surgery.^15^

Due to the relationship between age, growth, and development, early intervention methods cannot be applied to treat skeletal deformities in the permanent dentition or in adults. From this perspective, the only non-surgical alternative to manage skeletal deformities in the permanent dentition or in the adult is comprehensive treatment with fixed appliances.^8^

Nongrowing patients with moderate skeletal Class III malocclusion and acceptable facial esthetics can benefit from orthodontic camouflage,^16^ especially in cases of mild to moderate skeletal discrepancies.^11^ In the present case, which involved an adult patient with problems in the three planes (anteroposterior, vertical, and transverse) and indication for ortho-surgical treatment (option refused by the patient), orthodontic camouflage was chosen even with the limitations imposed by this choice (skeletal problems would not be corrected).

Despite the dissatisfaction with his smile esthetics, the patient showed a good appearance and was not bothered by his profile, which influenced his emphatic negative position regarding surgical correction. The decision for orthognathic surgery is mainly related to the self-perception of patients.^17^ Although dental specialists may recommend surgical treatment, self-perceptions of the facial profile are more important in the patient’s decision to choose this type of treatment.^9^

The results obtained with orthodontic camouflage were already expected. Dental compensations were performed to compensate for the existing discrepancy in the maxillary and mandibular bases, with the objective of restoring the function and providing some esthetical improvement, maintaining the initial inclination of the maxillary incisors and retroclination of the lower incisors. Troy et al.^3^ analyzed Class III patients treated with camouflage and orthognathic surgery, and compared the dental and skeletal results obtained. The results of camouflage treatment did not differ from those of our study: there were no skeletal changes, the maxillary incisors were proclined, and the mandibular incisors were retroclined.

There was good control of the vertical dimension, which was already increased, and no skeletal or profile changes. Other studies showed similar results, corroborating the indication of this treatment in cases of mild to moderate skeletal Class III malocclusion.^1^ ^,^ ^3^ ^,^ ^10^

The decision for orthodontic camouflage in the present case was made by considering some important parameters. First, the skeletal Class III malocclusion was mild, with ANB = 0 and little impairment of facial esthetics, which was irrelevant for the patient. Moreover, the anteroposterior position and the initial inclination of the maxillary and mandibular incisors were satisfactory for correcting the malocclusion, and the thickness of mandibular symphysis allowed good retraction of the mandibular incisors. When associated, all these factors provided greater safety for choosing this type of treatment.^10^ ^,^ ^11^

**Figure 7:**
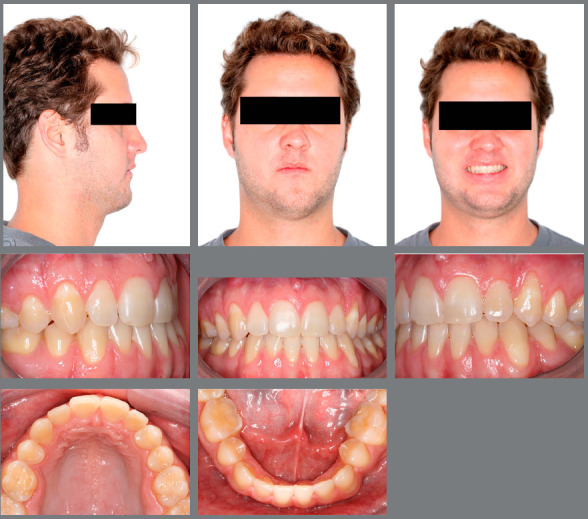
Facial and intraoral photographs 8 years after treatment

The retroclination of mandibular incisors in the camouflage treatment can result in prominent (vestibular) roots and gingival recessions. Therefore, care must be taken to attain a proper dentoskeletal relationship, especially in cases of severe skeletal dysplasias.^3^ Accordingly, the present patient was treated with great care, as he already showed significant gingival recession in tooth #33. The problem was monitored throughout treatment, and periodontal care was recommended, with rigorous dental plaque control. Canine distalization and incisor retraction were carefully performed, to maintain the normal gingival insertion levels of the incisors and prevent the increase of recession in tooth #33. Results showed that these procedures were effective, continuing throughout the retention phase (Figs 4 and 7).

**Figure 8:**
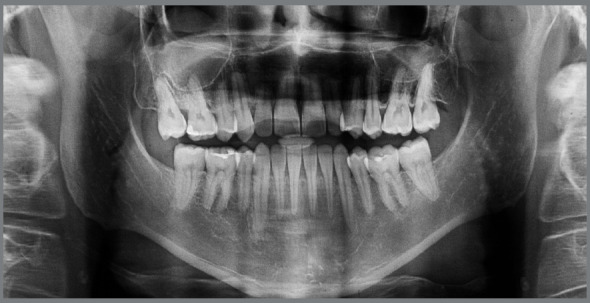
Panoramic radiograph 8 years after treatment.

**Figure 9:**
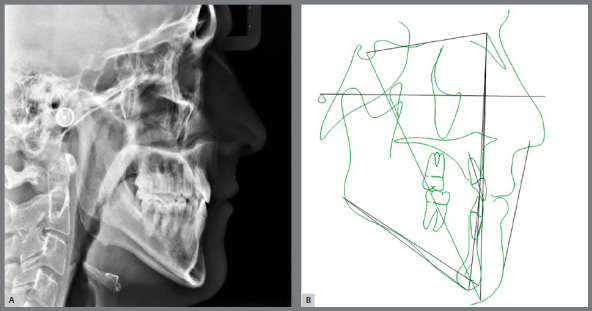
Cephalometric profile radiograph (A) and cephalometric tracing (B) 8 years after treatment.

**Figure 10:**
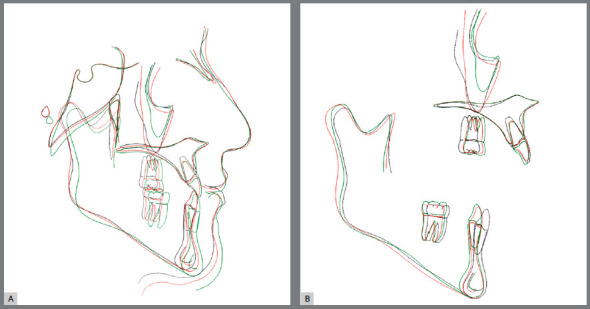
Initial (black), final (red) and 8 years after treatment (green) total (A) and partial (B) superimpositions of cephalometric tracings.

It is worth highlighting the need for a long-term follow-up to control the stability of the results obtained after retracting the mandibular incisors in patients with Class III malocclusion. Considering that the mandibular incisors are retracted by 4-5 mm, the tongue has a reduced space after treatment, resulting in increased pressure on these teeth, creating spaces between them. To prevent this, ideal overjet, overbite, and intercuspation should be sought, achieving upright mandibular posterior teeth after distalization, using lower retention, and, in cases of lingual interposition at rest or during deglutition, recommending multifunctional therapy.^11^ The patient did not seek the recommended services to remove preexistent deleterious habits. Nevertheless, his records for the retention phase, eight years after treatment, showed stability.

The results obtained were greatly valued by the patient, who returns annually for check-ups by his own initiative. Although not having an initially unfavorable facial esthetics, his smile bothered him. The improvement in the maxillary arch shape decreased the buccal corridor, resulting in better smile esthetics. Moreover, the elimination of mandibular crowding also contributed to these satisfactory results, with these being the chief complaints reported by the patient. The esthetic improvement resulting from malocclusion treatment enhances the oral health-related quality of life, especially for decreasing the psychological discomfort^18^ ^,^ ^19^.

Significant changes in the teeth and soft tissues can be expected in young Class III patients treated with orthodontic camouflage. A wide range of skeletal dysplasias can be camouflaged by dental movement without deleterious effects to the periodontium. For that purpose, diagnosis and treatment objectives should be realistically defined to prevent undesirable sequelae.^1^

## CONCLUSION

Orthodontic camouflage can be an effective treatment alternative for achieving functional occlusion, stability, and satisfactory esthetics in adult patients with mild to moderate skeletal Class III malocclusion.
